# Single-cell analysis of Kaposi’s sarcoma-associated herpesvirus infection in three-dimensional air-liquid interface culture model

**DOI:** 10.1371/journal.ppat.1010775

**Published:** 2022-08-17

**Authors:** Kyle L. Jung, Un Yung Choi, Angela Park, Suan-Sin Foo, Stephanie Kim, Shin-Ae Lee, Jae U. Jung

**Affiliations:** 1 Department of Cancer Biology and Global Center for Pathogen Research and Human Health, Lerner Research Institute, Cleveland Clinic, Cleveland, Ohio, United States of America; 2 Department of Molecular Microbiology and Immunology, Keck School of Medicine, University of Southern California, Los Angeles, California, United States of America; Hannover Medical School, GERMANY

## Abstract

The oral cavity is the major site for transmission of Kaposi’s sarcoma-associated herpesvirus (KSHV), but how KSHV establishes infection and replication in the oral epithelia remains unclear. Here, we report a KSHV spontaneous lytic replication model using fully differentiated, three-dimensional (3D) oral epithelial organoids at an air-liquid interface (ALI). This model revealed that KSHV infected the oral epithelia when the basal epithelial cells were exposed by damage. Unlike two-dimensional (2D) cell culture, 3D oral epithelial organoid ALI culture allowed high levels of spontaneous KSHV lytic replication, where lytically replicating cells were enriched at the superficial layer of epithelial organoid. Single cell RNA sequencing (scRNAseq) showed that KSHV infection induced drastic changes of host gene expression in infected as well as uninfected cells at the different epithelial layers, resulting in altered keratinocyte differentiation and cell death. Moreover, we identified a unique population of infected cells containing lytic gene expression at the KSHV *K2*-*K5* gene locus and distinct host gene expression compared to latent or lytic infected cells. This study demonstrates an *in vitro* 3D epithelial organoid ALI culture model that recapitulates KSHV infection in the oral cavity, where KSHV undergoes the epithelial differentiation-dependent spontaneous lytic replication with a unique cell population carrying distinct viral gene expression.

## Author summary

While KSHV is the etiologic agent of Kaposi’s sarcoma that frequently occurs in AIDS patients, there is not biologically relevant, reproducible *in vitro* culture system to study KSHV infection and spontaneous lytic replication. Here, we describe that a 3D human oral epithelial ALI culture that can support a robust KSHV infection and spontaneous lytic replication associated with oral epithelial differentiation. scRNAseq analysis identified considerable alteration of host gene expression in both KSHV-infected cells and -uninfected bystander cells. Our study provides insights into the early stages of KSHV infection in oral epithelial cells and serves as a useful blueprint for the investigation of KSHV infection and transmission.

## Introduction

KSHV is a gamma herpesvirus and the etiologic agent of Kaposi’s sarcoma (KS), primary effusion lymphoma, and multicentric Castleman’s disease [1–3]. Like other herpesviruses, KSHV switches between the latent and lytic lifecycles [4]. Latent KSHV is characterized by limited viral gene expression, dependency on the host for replication of the viral episome, and co-segregation of the viral episome with host chromosomes during mitosis. KSHV defaults to this quiescent, latent phase to evade the host immune system and maintain a persistent, lifelong infection. In response to an external stimulus such as oxidative stress, hypoxia, and certain chemicals, the latent KSHV lifecycle can be disrupted and enter lytic reactivation [5].

The oral cavity provides a favorable environment for the virus to reactivate upon external stimuli. Previous studies have shown that several infectious pathogens, including *Staphylococcus aureus*, stimulate lytic reactivation in KSHV-infected oral cells through their pathogen-associated molecular patterns (PAMPs) and host Toll-like receptor (TLR) signaling [6]. Moreover, intensive epigenetic reorganization during oral epithelial differentiation may trigger KSHV reactivation [7]. In addition, the oral cavity sheds infectious KSHV virions into the saliva two to three log folds higher in titer compared to other anatomic sites [8], which is why oral transmission is considered as the major route of KSHV transmission [9]. However, the molecular mechanism behind how KSHV establishes infection in the oral cavity and how lytic reactivation is triggered in the oral epithelial cells remains unclear.

The oral buccal mucosal epithelium serves as a barrier between the underlying oral tissues and the outside environments such as the soft palate, cheeks, and floor of the mouth [10]. The composition of human oral epithelial barrier is complex, with a structure consisting of three major layers: basal, intermediate, and superficial. The basal layer consists of mitotically active epithelial cells connected to neighboring cells through desmosomes at the bottom of the epithelium [11]. These cells are characterized by the expression of *Keratin 5* (*KRT5*), *Keratin 14* (*KRT14*), and *S100A2* [12–14]. As the basal cells differentiate upwards into the intermediate layer, they downregulate the expression of *KRT5*, *KRT14*, and *S100A2*, and express a new set of genes such as *Keratin 13* (*KRT13*), *NOTCH3*, *S100A4*, and *TGM5* [11,12,14–16]. As these cells differentiate into the superficial or cornified layer, they undergo another major transcriptional change in order to deposit glutamine- and lysine-rich proteins such as SPRR2A and SPRR2E [15] on the inner surface of the plasma membrane to be crosslinked with the keratin cytoskeleton with an influx of Ca^2+^ that also upregulates S100 proteins such as S100P [14]. These three layers ultimately form oral epithelial tissues for the protection of the organism from chemical, microbial, and physical challenges.

While 2D monolayer cultures of oral epithelial cell-based infections have been extensively studied, they are limited by the lack of physiological relevance in mimicking the epithelial barrier. A deeper understanding of KSHV *de novo* infection and its spontaneous reactivation require comprehensive characterization of virus infection in multiple epithelial cell subtypes. *In vitro* epithelial air-liquid interface (ALI) culture models are highly relevant tools that are able to complement animal models for understanding the pathological mechanism of KSHV infection [17]. While these ALI models mimic human oral epithelial tissue and demonstrate KSHV reactivation during epithelial cell differentiation [17,18], *de novo* infection of KSHV in this model has not been reported.

Using 3D oral epithelial ALI cultures fully differentiated from healthy oral keratinocytes, we developed a novel *de novo* KSHV infection model. We found that exposing the basal layer was necessary for initial KSHV infection and confirmed that epithelial differentiation tightly correlated with KSHV lytic reactivation. To further understand the effect of KSHV on host gene expression in oral epithelial tissues, we leveraged scRNAseq to profile the gene expression across all cells in the different epithelial layers. We identified that host gene expression in infected cells, as well as uninfected cells, were drastically changed by presence of KSHV, thereby altering keratinocyte differentiation and cell death. Moreover, we discovered a distinct population of infected cells with a limited early lytic KSHV gene expression and unique host gene expression profile compared to latent or lytic cells. Taken together, our proposed *de novo* KSHV infection model demonstrates a physiologically relevant model that closely mimics KSHV infection in the oral cavity and provides insight into the early stages of KSHV infection in oral transmission.

## Results

### KSHV infection of primary oral epithelial ALI culture undergoes spontaneous replication

**Fig 1:**
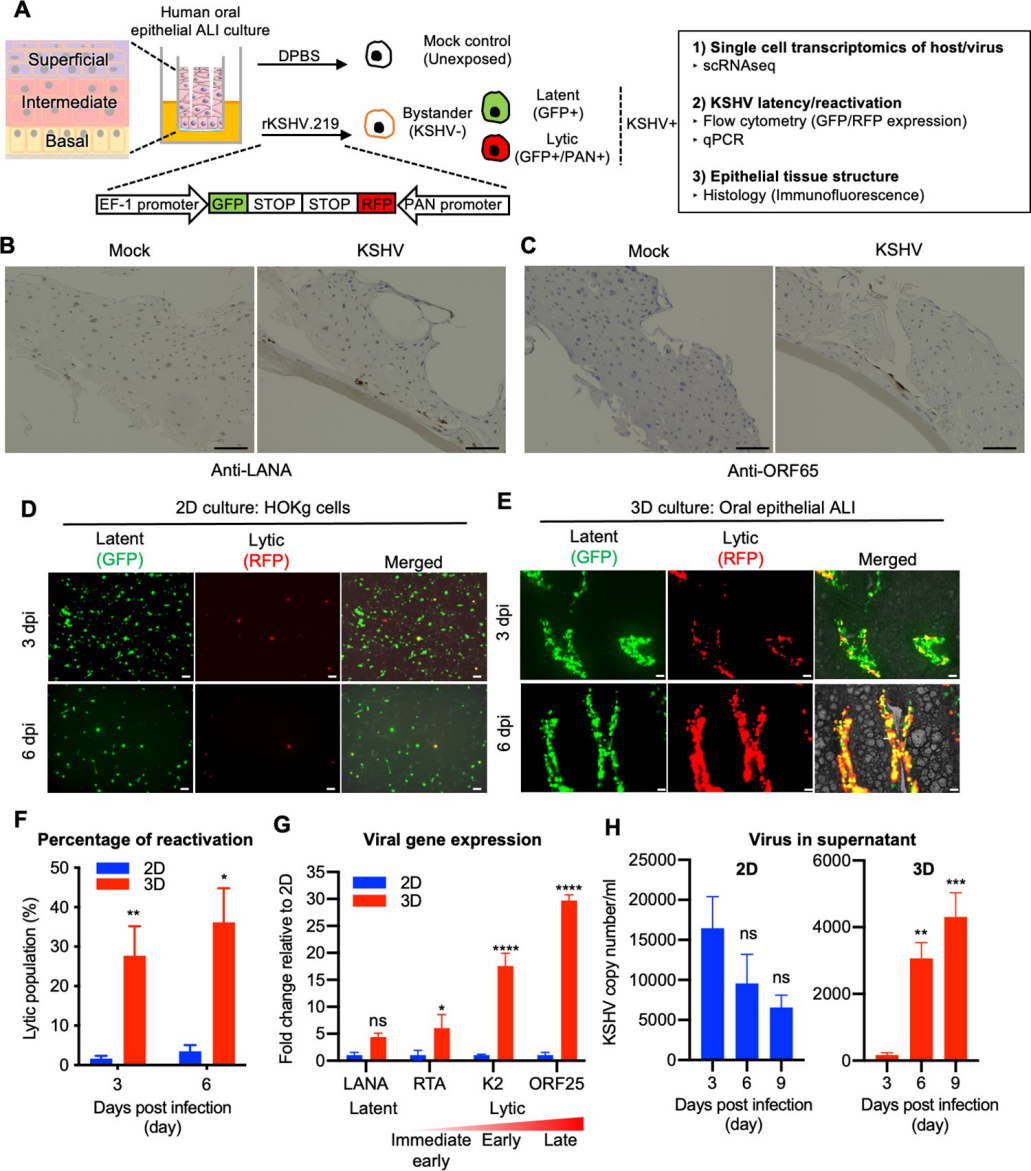
KSHV infection of primary oral epithelial ALI culture model shows more spontaneous reactivation than 2D monolayer culture. (A) Schematic of the experiment. Human 3D oral epithelial tissues consisting of basal (gold), intermediate (red), and superficial (purple) layers were wounded to expose the basal layer epithelial cells. The tissues where then infected with either DPBS or rKSHV.219 and harvested either 3 or 6 days post infection (dpi). Uninfected cells from the mock tissue are labeled as unexposed cells while uninfected tissues from the tissues exposed to rKSHV.219 are labeled as bystander cells. All cell types were used for 3 main studies: single cell transcriptomics of host and viral genes, KSHV latency and reactivation, and epithelial tissue structure. Fig 1A was created with a modified image from Servier Medical Art, which is licensed under a Creative Commons Attribution 3.0 Unported License (https://creativecommons.org/licenses/by/3.0/). Immunohistochemistry of KSHV LANA (B) or ORF65 (C) in mock or KSHV infected tissues at 6 dpi. Bars = 100 μm. (D) GFP and RFP expression in cells infected with rKSHV.219 for 3 days and 6 days in HOKg 2D culture cells and (E) 3D epithelial tissues. The images are taken from above the cell cultures. Bars = 200 μm. (F) Lytic population (Percentage of RFP+ and GFP+ expressing cells in GFP+ cells) were detected by FACS. (G) Quantification of LANA, (latency), RTA, (immediate early lytic), K2, ORF25 (early lytic), ORF25 (late lytic) mRNA levels in HOKg 2D culture cells and in 3D epithelial tissue infected with rKSHV.219 at 3 dpi. (H) KSHV genome copy number in supernatant of HOKg 2D culture cells (left) and 3D epithelial cultures (right) infected with rKSHV.219 at the indicated time point.

3D oral epithelial tissues in ALI culture are 8–11 cells thick with differentiated basal, intermediate, and superficial epithelial layers. Intact 3D oral epithelial tissues were initially infected from the apical side with rKSHV.219 that contains green fluorescent protein (*GFP*) gene indicating KSHV infection and red fluorescent protein (*RFP*) gene under the control of the PAN RNA promoter indicating KSHV lytic replication [19] (Fig 1A). Since KSHV failed to establish a productive infection in an intact 3D oral epithelial tissue, we introduced scoring to disrupt the epithelial barrier with (deep) or without (light) basal exposure for KSHV infection (S1A Fig). When these wounded tissues were incubated with either Dulbecco’s phosphate-buffered saline (DPBS, Mock) or infectious rKSHV.219, KSHV was able to establish an infection upon deep wounding-induced exposure of the basal epithelial cells (S1B Fig). Immunohistochemistry and immunofluorescence assays also revealed that cells around wounded area expressed LANA and ORF65 (Fig 1B and 1C) and that GFP+ and RFP+ cells were co-stained with LANA and ORF59, indicating GFP and RFP signals as surrogate markers for KSHV infection (S1C Fig). Interestingly, KSHV entry receptors integrin β1 and CD98 were primarily expressed in the basal epithelial cells (S1D Fig), suggesting that integrin β1 and CD98 expression in the basal layer allows efficient KSHV infection [20,21].

While primary normal human oral gingival keratinocytes (HOKg) were highly susceptible to KSHV infection (GFP+) in 2D culture, only a minor population [~3% at 6 days post infection (dpi)] of KSHV-infected cells underwent spontaneous lytic replication (RFP+) (Figs 1D, 1F and S2). In contrast, a large portion (~45% at 6 dpi) of KSHV infected basal epithelial cells in 3D organoids underwent spontaneous lytic replication (RFP+) (Figs 1E, 1F and S2). Moreover, KSHV-infected 3D epithelial cultures showed significantly increased expression of *RTA* (immediate early), *K2* (early), and *ORF25* (late) across all lytic stages (Fig 1G). Finally, while viral copy number decreased over time in the supernatants of 2D culture (Fig 1H, *left*), it rapidly increased in the supernatants of 3D tissue culture, suggesting virion release (Fig 1H, *right*). This study of primary oral 3D epithelial ALI cultures demonstrates that KSHV readily infects the basal epithelial layers induced by deep wounding and undergoes a rapid transition from latency to spontaneous lytic replication to potentially release virions, which may recapitulate *in vivo* human oral KSHV infection.

### Single-cell RNA sequencing reveals enriched lytic KSHV-infected cells in the superficial epithelia

**Fig 2:**
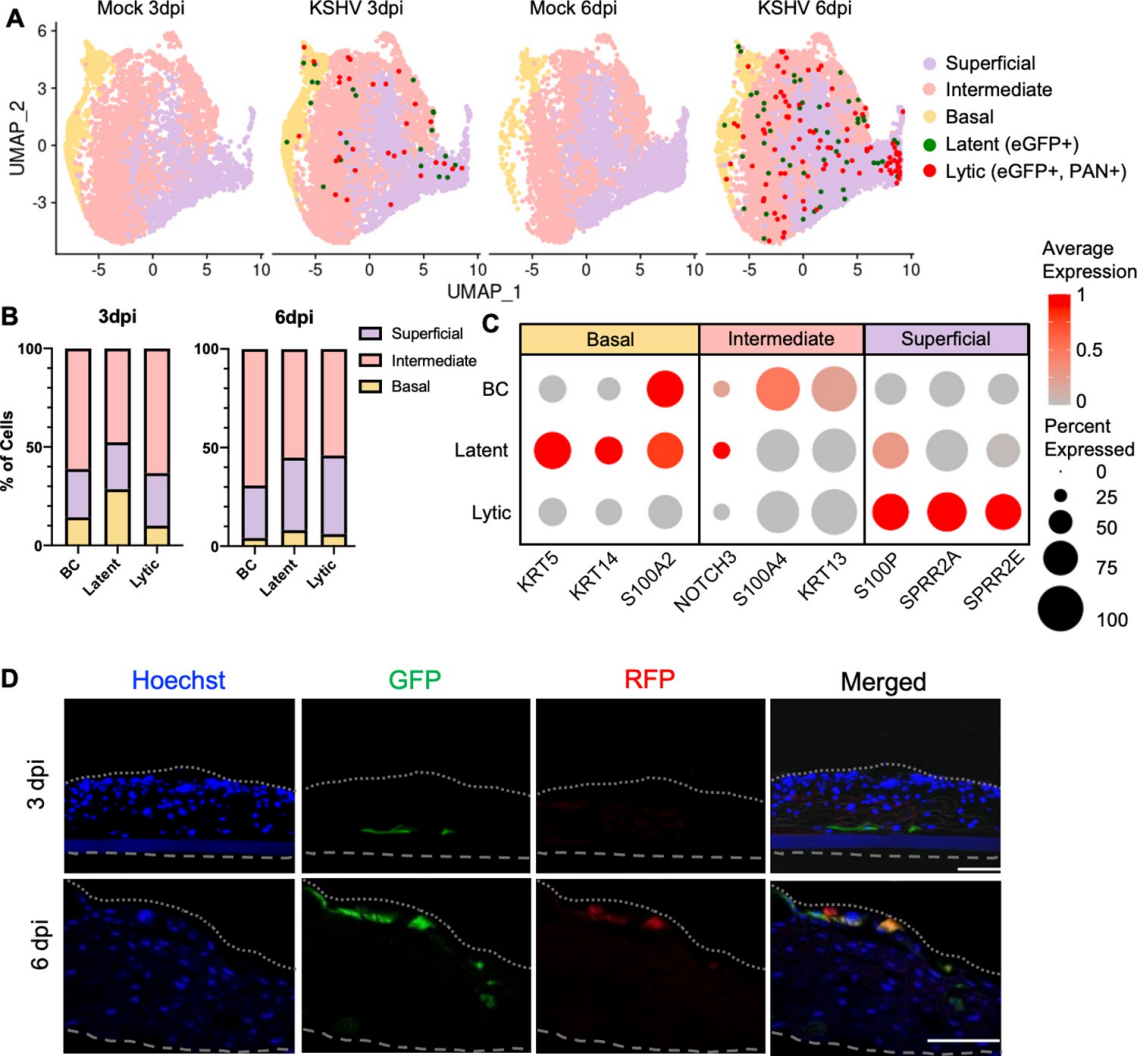
Single-cell RNA sequencing reveals that lytic KSHV cells are enriched in the superficial epithelia. (A) Uniform manifold approximation and projection (UMAP) of single cells harvested at either 3 or 6 dpi. Cells from KSHV infected 3D epithelial tissues and mock controls colored by either epithelial layer (basal, intermediate, or superficial) or KSHV lifecycle (latent or lytic). (B) Bar charts show the percentage of bystander (BC), latent, or lytic cells in each epithelial layer of the infected tissue at 3 or 6 dpi. (C) Dotplot showing the expression gene expression markers from the basal, intermediate, and superficial layers of the epithelia. The size of each dot corresponds to the percentage of cells expressing the gene within bystander (BC), latent or lytic infected cells. The color of each dot represents the average expression level for each gene within each population. (D) Immunofluorescence images of Hoechst staining, GFP, or RFP expression within the infected 3 dpi or 6 dpi ALI epithelial tissues. The dashed gray lines indicate the bottom and top of the 3D epithelial tissues. Bars = 100 μm.

To gain a better understanding of KSHV-infected cells undergoing spontaneous reactivation, we performed scRNAseq on infected 3D epithelial tissues. 3D epithelial tissues established apparent infection around 3 dpi and increased spontaneous reactivation throughout the culture (S3A Fig). Since 3D oral epithelial tissues lost structural integrity at 9 dpi in either mock infection or KSHV infection condition, we selected 3 and 6 dpi of 3D epithelial tissues for scRNAseq analysis. After quality control to remove dying cells (S4A–S4C Fig), we analyzed 36,144 cells harvested from KSHV-infected 3D epithelial tissues and their respective mock controls at 3 and 6 dpi from three independent sets of infection. We used gene expression markers to define the basal layer (*KRT5*, *KRT14*, *S100A2*), intermediate layer (*NOTCH3*, *S100A4*, *KRT13*, *TGM5*), and superficial layer (*S100P*, *SPRR2A*, *SPRR2E*). Unsupervised clustering showed that cells clustered based on epithelial layer (basal, intermediate, or superficial) and showed progression of epithelial cell differentiation (Fig 2A).

Since the 10X genomics single cell gene expression platform has a limited RNA capture rate of ~65%, low abundant viral transcripts such as LANA and RTA were mostly undetected. For this reason, we used GFP and RFP expression as surrogate markers to distinguish the population of virus-infected cells within the infected tissues: for instance, GFP+ and GFP- cells were designated as KSHV-infected cells and bystander cells, respectively (Fig 1A). Since PAN RNA is the most abundant viral transcript during lytic cycle [22,23], PAN RNA expression was used to further define GFP+/PAN- latent infected and GFP+/PAN+ lytic infected cells (Fig 2A–2C). This showed an enrichment of latent cells in the basal layer (28.57%) at 3dpi and of latent and lytic cells in the superficial layer (36.7% and 39.8%, respectively) at 6dpi (Fig 2B). Lytic cells also expressed higher levels of the superficial layer-specific genes *S100P*, *SPRR2A*, and *SPRR2E* (Fig 2C). In the cross section of 3D epithelial tissues, GFP+/RFP+ cells were in the superficial layer, while GFP+/RFP- cells were mainly in the basal layer (Fig 2D). In addition, lytic protein ORF59- and K8.1-expressing lytic cells were mostly present in the superficial exposed areas, while LANA-expressing latent cells were primarily in the basal layer (S3B Fig). This indicated that *de novo* infection of KSHV occurred only in the basal layer and that KSHV underwent spontaneous reactivation in the superficial layer, where lytic replicating cells were enriched.

### KSHV infection dysregulates epithelial differentiation

**Fig 3:**
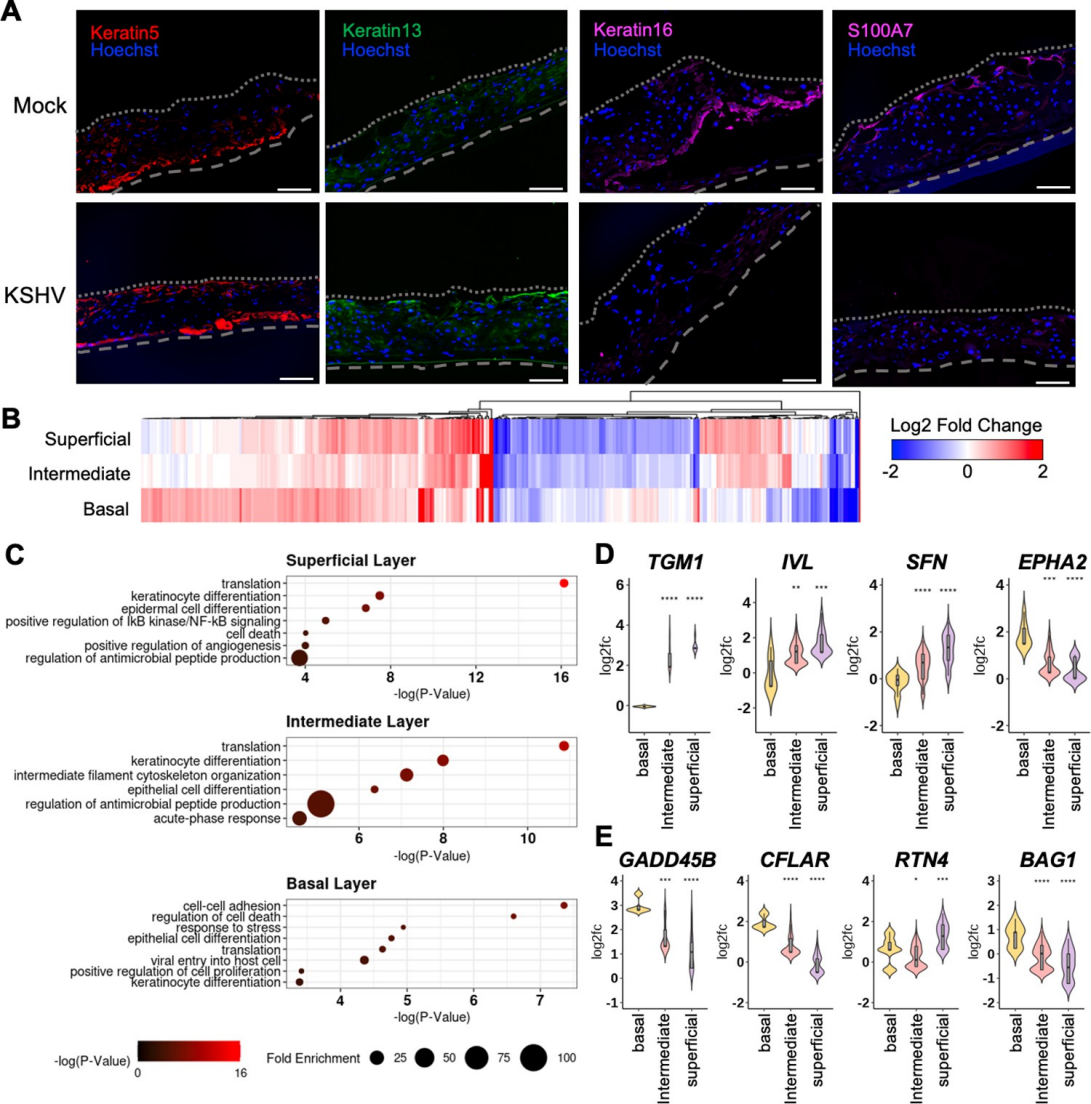
KSHV infection dysregulates host epithelial differentiation. (A) Immunofluorescence images of mock- or KSHV-infected 3D oral tissues. Keratins 5 is stained red and keratin 13 is stained green while keratin 16 and S100A7 are stained purple. Hoechst nuclei staining (blue) in all pictures. Bars = 100 μm. (B) Heatmap of significantly upregulated and downregulated genes in all KSHV-infected cells compared to mock cells in each epithelial layer (3 and 6 dpi combined). Significant genes have a |fold change| > 1.5 and p-value < 0.05. (C) Gene ontology analysis of pathways affected in KSHV-infected cells compared to mock cells from each epithelial layer. The color of each dot represents the -log_10_(p-value) and the size of each dot represents the fold enrichment (observed # of genes/expected # of genes). Violin plots for genes related to keratinocyte differentiation (D) and cell death (E). For each violin plot, the log2 fold change is shown comparing KSHV-infected cells and mock-infected cells within each epithelial layer. Boxplots in gray within the violin show the first quartile, median, and third quartile.

To characterize epithelial cell differentiation upon KSHV infection, we used immunofluorescence to visualize the epithelial differentiation in mock and KSHV-infected 3D oral epithelial ALI cultures (Fig 3A). As keratin 5 and keratin 13 are the basal and suprabasal layer markers, respectively [24], keratin 5 was exclusively detected in the basal layer epithelia and keratin 13 was detected throughout the suprabasal layer of mock-infected 3D cultures. In contrast, keratin 5 and keratin 13 were expressed in the basal, suprabasal and superficial epithelial layers in KSHV-infected 3D cultures. Furthermore, since expression of keratins 16 and S100A7 are induced in response to tissue wounding [25,26], their expressions were readily detected in mock-infected 3D culture but weakly detected in KSHV-infected 3D cultures (Fig 3A). These data suggest that KSHV infection apparently alters epithelial differentiation and wound healing pathways.

scRNAseq analysis identified that host gene expression within each epithelial layer was significantly altered upon KSHV infection compared to mock infection (Fig 3B). Pathway analysis showed that while KSHV infection commonly affected keratinocyte differentiation and protein translation throughout all epithelial layers, it uniquely altered specific pathways at each layer: cell-cell adhesion and response to stress in the basal layer; intermediate filament cytoskeleton organization and acute-phase immune response in the intermediate layer; and cell death and angiogenesis in the superficial layer (Fig 3C). The intermediate and superficial layers of KSHV-infected tissues showed the upregulated expression of *TGM1*, *IVL*, and *SFN* genes that promote keratinocyte differentiation and the downregulated expression of *EPHA2* gene that restricts keratinocyte differentiation (Fig 3D). Finally, the superficial layer of KSHV-infected tissues showed the upregulated expression of apoptosis inducer *RTN4* [27] and the downregulated expression of apoptosis suppressors *GADD45B*, *CFLAR*, and *BAG1* (Fig 3E). This indicates that KSHV infection dysregulates epithelial differentiation pathway throughout all three layers and host signaling pathways of each layer in a unique way.

### Effect of KSHV infection on gene expression of basal epithelia in 3D organoids

**Fig 4:**
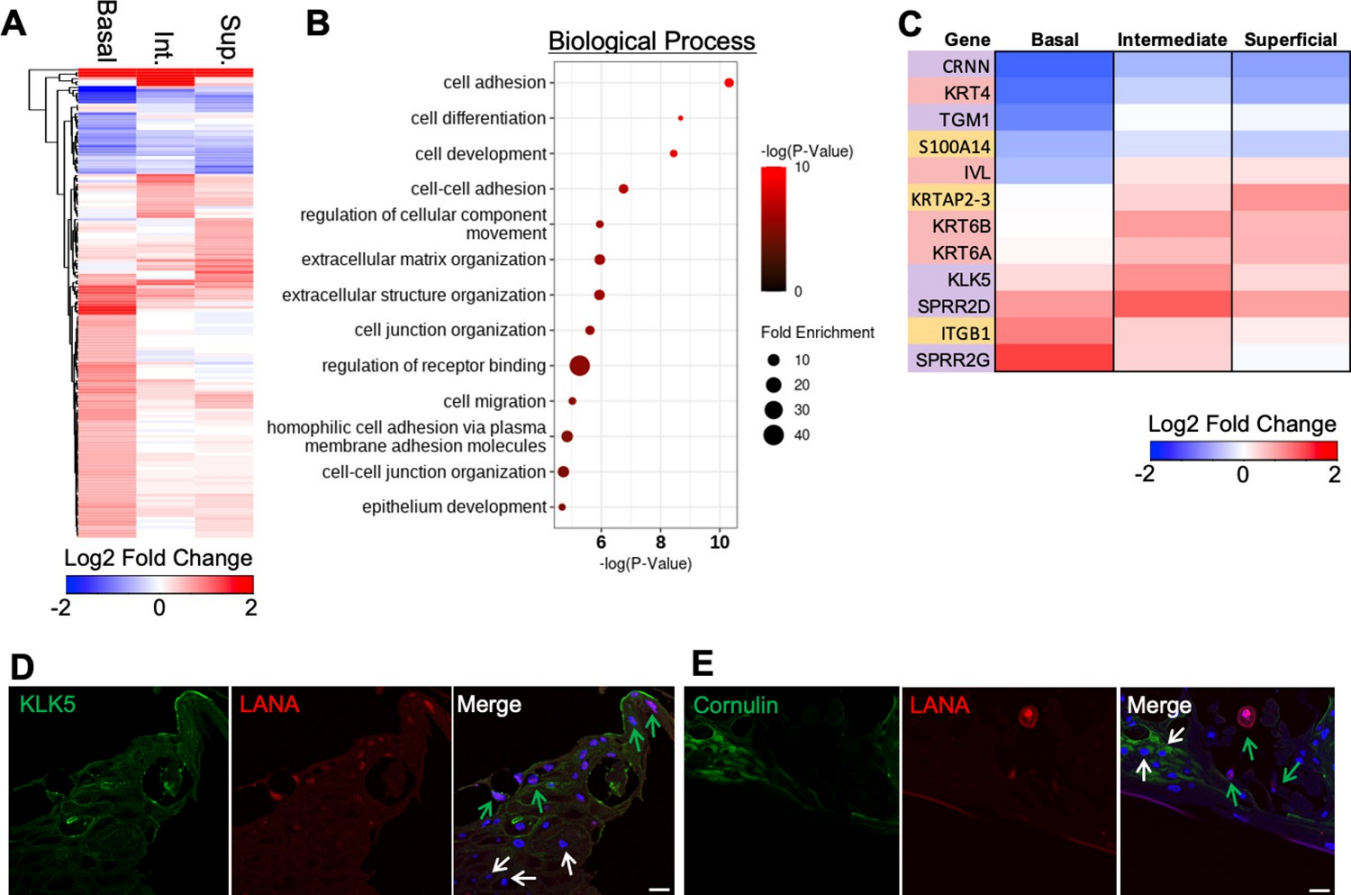
KSHV predominantly affects basal epithelial cells in KSHV-infected tissues. (A) Heatmap of significantly upregulated and downregulated genes in all KSHV-infected cells compared to bystander cells within each epithelial layer (3 and 6 dpi combined). Significant genes have a |fold change| > 1.5 and p-value < 0.05. (B) Gene ontology analysis of the top pathways affected by KSHV infection within basal epithelial cells. This analysis compares basal KSHV-infected cells to bystander cells with the color of each dot representing the p-value and the fold enrichment (observed # of genes/expected # of genes). (C) Heatmap of keratinocyte differentiation related genes. Basal layer genes are highlighted in gold, intermediate layer genes are highlighted in red, and superficial layer genes are highlighted in purple. Log2 fold change represents the average fold change when comparing KSHV-infected cells and bystander cells within each epithelial layer. Co-staining of KLK5 and LANA or (D) cornulin and LANA (E) in 3D oral epithelial cultures at 6 dpi with overlay of Hoechst staining (blue). LANA+ KSHV infected cells are indicated by green arrow. LANA- bystander cells are indicated by white arrow. Scale bar = 25 μm.

When KSHV-infected cells were compared to the uninfected bystander cells in infected 3D epithelial ALI culture, KSHV infection induced more pronounced alterations of cellular gene expressions in the basal layer than in the intermediate or superficial layer (Fig 4A). Specifically, KSHV infection in the basal layer led to the upregulation of several host signaling pathways including cell adhesion, cell differentiation, and extracellular matrix organization (Fig 4B). When keratinocyte differentiation-related genes were analyzed within each layer of epithelial organoids upon KSHV infection, they were dramatically dysregulated in KSHV infected cells compared to those in bystander cells (Fig 4C). Specifically, KSHV infected cells highly expressed *kallikrein-5* (*KLK5*) that is involved in the terminal differentiation of keratinocytes, while they showed lower expression of *cornulin* (*CRNN*) that is a marker of squamous cells (Fig 4C). Furthermore, immunofluorescence assay also confirmed that compared bystander cells, LANA-positive cells showed higher expression of KLK5, but lower expression of CRNN (Fig 4D and 4E). Loss of CRNN expression has been associated with advanced tumor stage, whereas high expression of KLK5 has been positively correlated with tumor invasion [28,29]. This indicates that KSHV infection-mediated alteration of host gene expressions in 3D epithelial ALI culture potentially contributes to viral cancer progression.

### A unique cell population with a distinct KSHV gene expression profile

**Fig 5:**
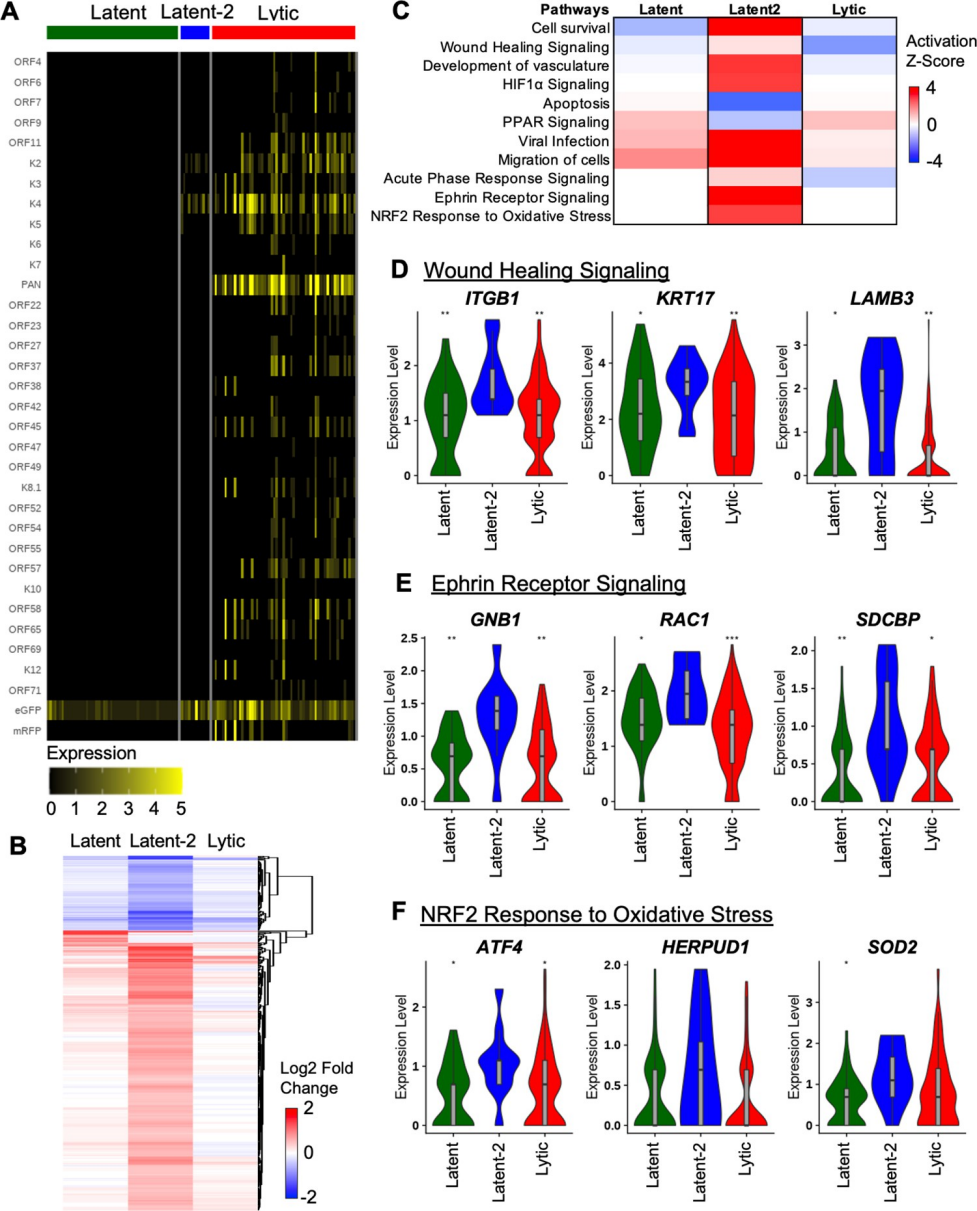
KSHV latent-2 cells are a unique viral population with a distinct gene expression profile. (A) Heatmap of viral gene expression in KSHV cells (3 and 6 dpi combined). Latent cells are GFP+. Latent-2 cells are GFP+/PAN- with gene expression from the K2 to K5 area of the genome. Lytic cells are GFP+/PAN+ cells. (B) Heatmap showing all significantly expressed genes in latent, latent-2, and lytic cells. Expression is shown as log2 fold change comparing latent, latent-2, and lytic cells to the bystander cells. (C) Heatmap showing pathway activation z-scores of the KSHV cell populations compared to mock cells. (D-F) Violin plots showing gene expression of genes involved in the wound healing, ephrin receptor signaling, or NRF2 response to oxidative stress. Statistical comparisons are compared to the latent-2 population. * indicates p-value ≤ 0.05, ** indicates p-value ≤ 0.01, *** indicates p-value ≤ 0.001. Boxplots in gray within the violin show the first quartile, median, and third quartile.

As KSHV exhibits a biphasic lifecycle between latent and lytic infection, we separated KSHV-infected cells into latent (GFP+/PAN RNA-) and lytic cells (GFP+/PAN RNA+ or RFP+) to examine their viral gene expressions (Fig 5A). During *de novo* infection, approximately 39% and 26% of infected cells showed latency at 3 dpi and 6 dpi, respectively, while 58% of infected cells spontaneously underwent lytic reactivation at 3 dpi and this lytic cell population increased to 66% at 6 dpi (S1 Table). Since the numbers of KSHV-infected cells were low, we combined 3 and 6 dpi timepoints for analyses relating to viral life cycle with the rationale that viral infection stage is the defining factor of infected cells. Interestingly, we found a unique population within the latent group that contained specific lytic gene expression at the KSHV *K2*-*K5* locus and designated these cells as “latent-2” cells (Figs 5A and S3C). Previous work has shown that activating histone modifications are enriched at the *K2*-*K5* locus, accompanied by the transient induction of limited lytic gene expression [30]. The latent-2 population was 1.9% of infected cells at 3 dpi and expanded to 6.8% of infected cells at 6 dpi (Fig 5A, S1 Table). Heatmap analysis of 3 and 6 dpi cells showed that latent-2 cells carried distinct host gene expression pattern from latent and lytic cells (Fig 5B). Pathway analysis showed that a few signaling pathways including viral infection and migration of cells were similarly affected in latent, latent-2, and lytic cells compared to mock-infected cells (Fig 5C). On the other hand, latent-2 cells showed different profiles of several signaling pathways from latent or lytic cells: cell survival, wound healing, HIF1α signaling, ephrin receptor signaling, and NRF2 response to oxidative stress were upregulated but apoptosis and PPAR signaling were downregulated (Fig 5D–5E). A recent Landis *et al* paper [31] also has demonstrated distinct latent lifecycle of KSHV in JSC-1 and BCBL-1 PEL cells that carries a latent-2 like viral gene expression pattern at the KSHV *K2* gene region. This reveals the heterogenous nature of KSHV gene expression in oral epithelial 3D organoid upon *de novo* infection and latent-2 cell population shows a distinct host gene expression profile from latent or lytic cell population.

## Discussion

The oral cavity is the major route for KSHV infection and exposure to saliva containing infectious KSHV is primarily responsible for the transmission [8]. Thus, understanding KSHV infection of the oral cavity is essential for the study of viral transmission and pathogenesis. Here, we described a *de novo* KSHV infection model of fully differentiated 3D oral epithelial ALI culture that appears to fully recapitulate *in vivo* oral KSHV infection.

Although KSHV is orally transmitted, viral transmission in the oral epithelia is not well understood. Previous studies have shown that KSHV enters cells by actin-mediated micropinocytosis in endothelial cells or clathrin-mediated endocytosis in fibroblasts [32,33]. CD98, integrins α3β1, αVβ3, αVβ5 and EphA2 receptor have been reported as the initial binding partners of KSHV to the cell surface [20,34,35]. Here, we found that the apical surface of intact oral epithelium was resistant to KSHV infection unless we introduced wounding to provide access to the basal layer of oral epithelial tissue. This could be due to a lack of host receptors for KSHV binding or entry at the apical membrane of differentiated tissues since CD98 and Integrin ß are mainly expressed on the basal layer. It is also possible that wounding causes the disruption of physical barrier like tight junction at the superficial layer to allow KSHV infection. Nevertheless, this suggests that KSHV infects the oral epithelia in a manner similar to human papilloma virus [36,37], where infectious virions also require access to basal epithelial cells.

Previous transcriptome analyses of KSHV-infected cells or KSHV-associated tumors have been based on bulk RNAseq study, which disregards cell and tissue heterogeneity [38,39]. However, KSHV infection is a diverse process with uninfected and infected cells along with different stages of viral infection. For example, latent infection and low-level reactivation coexist in KSHV-infected cells and tissues, and both are known to contribute to viral pathogenesis and tumorigenesis. Additionally, the oral epithelium, which is the initial infection site of KSHV, consists of multiple layers of basal, intermediate, and superficial cells. Given that multiple factors contribute to the establishment of KSHV infection, we leveraged the power of scRNAseq to profile gene expression of mock- and KSHV-infected oral epithelial tissues at a single cell level. Despite this strength, scRNAseq also has a number of limitations. The most significant limitation of the drop-seq single cell method is the low sensitivity [40] wherein low abundant viral transcripts are often undetected. For this reason, we used GFP and RFP as surrogate latent and lytic makers, respectively, to differentiate KSHV latent *vs*. lytic cells and identified the enrichment of latent and lytic cells at the superficial layer of infected tissues at 6dpi. This finding correlates with previous studies that show the presence of lytic cells at the superficial layers [17,18]. Our study also suggests that epithelial cell differentiation signaling and KSHV lytic replication are highly interconnected and potentially affect each other. Furthermore, we found that spontaneous lytic replication resulted in increased virion production over time in 3D oral epithelial tissues, which is also consistent with infectious KSHV particle production and transmission within the oral cavity [7,8,41]. These suggest that epigenetic changes during epithelial differentiation in KSHV-infected 3D epithelial cells may trigger spontaneous viral lytic gene expression, leading to infectious virion production.

KSHV replication not only requires epithelial differentiation, but also leads to the deregulation of epithelial differentiation in 3D oral epithelial ALI culture. Immunohistochemistry of KSHV-infected tissues showed that keratin 5 expression was induced in all layers, whereas S100A7 and keratin 16 expressions were considerably suppressed. scRNAseq also demonstrated that KSHV infection affected epidermal and keratinocyte differentiation, which might be linked with AP-1 activation induced by KSHV-mediated mitogen-activated protein kinase pathway [7,42]. Further sub-clustering of epithelial layers identified the distinctive effects of KSHV on host gene expression within each epithelial layer. For instance, the upregulation of basal layer cell proliferation was likely mediated by KSHV LANA [43,44] and the high lytic reactivation of KSHV at the superficial layer might lead to increased cell death. This suggests that KSHV infection displays distinctive effects on host gene expression in different epithelial layers, ultimately modulating its own viral lifecycle.

KSHV gene expression is used to classify viral lifecycle to latent, immediate early lytic, early lytic, and late lytic infection. To date, it is still unknown how KSHV gene expression is precisely regulated during *de novo* infection at a single cell level. Interestingly, we found a unique population of GFP+/RFP-/PAN- latently infected cells, designated “latent-2”, with specific gene expressions at the *K2*-*K5* locus. The previous studies [30,31] have also shown that similar to latent-2 cells, newly *de novo* infected cells and latently infected BCBL-1 and JSC-1 cells exhibit the transient lytic gene expressions at the *K2*-*K5* locus in 2D culture. It is possible that these latent-2 cells may be in transition from latency to lytic replication or represent the result of an abortive lytic infection following *de novo* infection. However, it should be noted that even in transitional state or abortive lytic infection, these cells carry distinctive, not random, profile of viral gene expression. In addition, latent-2 cells showed strikingly different phenotypes of various cellular signaling pathways from latent or lytic cells, including cell migration, ephrin receptor signaling, NRF2 response to oxidative stress, wound healing signaling, and apoptosis. Latent-2 cells also showed increased HIF1α signaling that has been previously implicated as a trigger for lytic reactivation [45]. Further study is warranted to investigate the details of latent-2 cells and their role in KSHV biology.

In summary, our study presents a novel KSHV infection model of 3D oral epithelial organoid ALI culture that allows the spontaneous replication and virion production as seen in oral lesions of KS patients. scRNAseq demonstrates that KSHV infection induces drastic changes of epithelial differentiation, which in turn contributes to viral lytic replication. Finally, the study also uncovers a unique population of infected cells with distinct host and viral gene expression. Thus, our *in vitro* 3D epithelial ALI culture model should be a valuable tool to further dissect KSHV infection and transmission at a basic science level and to screen for anti-viral therapeutics to block KSHV transmission at a public health level.

## Materials and methods

### Virus preparation and titration

KSHV infections were performed with recombinant strain KSHV.219 (rKSHV.219) prepared from induced iSLK.219 cells as described previously [46]. Seventy percent confluent iSLK.219 cells were induced with growth medium containing 1 mM sodium butyrate (Sigma Aldrich Cat. No. 303410-100G) and 1 μg/ml doxycycline (Sigma Aldrich Cat. No. D9891) in the absence of puromycin. After 3 days, the virus-containing culture media was collected and cleared by centrifugation and filtering to remove cells and debris. The virus was concentrated by ultracentrifugation for 3 hours at 24,000 rpm in SW32 rotor (Beckman Coulter). Virus pellets were resuspended in DPBS and stored at -80°C. Infectious virus amounts were determined and calculated by titration on primary normal human oral keratinocytes gingiva (HOKg) (Lifeline Cell Technology SKU: FC-0094) cells by measurement of GFP expression using a FACSCanto flow cytometer (BD Biosciences), assuming that one infectious unit (IU) generated one GFP-positive HOKg. Flow cytometry data were analyzed with the FlowJo software.

### De novo KSHV infection

EpiOral (ORL-606) tissues were purchased from MatTek and transferred into 6-well plates containing assay medium (GIN-100-ASY) for 1 hour upon arrival. The tissues are then scored with a blunted 31g insulin syringe (BD Cat. 305196). The level of scoring of EpiOral tissues was verified by microscope. Light scoring was determined by an intact layer of cells at the bottom of the wounding. Deep scoring can be determined by whether the insert membrane was exposed in the wounded area. The tissues are then infected on the apical side with 1 x 10^6^ IU of KSHV.219 for 4 hr. Following the infection, the tissues were washed with DPBS twice, returned to an air-liquid interface, and kept in 26 ml of assay medium in 10-cm dish. Tissues are incubated at 37°C for 3 or 6 days before harvesting while changing the media every 2 days.

For KSHV infection of 2D monolayer cultures, HOKg were purchased from LIFELINE and cultured in keratinocyte medium complete kit (Lifeline Cell Technology SKU: LL-0007). Cells were seeded one day before infection. When cell reached 60 to 70% confluence, 2 X 10^4^ IU of KSHV.219 were added to the cells to obtain an infection similar to the 3D culture infection. After incubation for 4 hours at 37°C, cells were washed with DPBS twice, followed by addition of growth medium before being returned to the incubator.

### Histology and immunofluorescence staining of ALI epithelial tissue

In order to determine the location of GFP and RFP expressing cells, the tissues were fixed with 10% formalin at room temperature for 1 hour, washed, and snap-frozen in Tissue-Tek O.C.T. Compound (Sakura Finetek USA Inc. 4583). Cryosections of 15 μm were cut and incubated with Hoechst 33342 and then examined by fluorescence microscope (Keyence BZ-X810). To stain for differentiation markers, 100% methanol was added to ALI sections for permeabilization and quenching GFP and RFP fluorescence for 10 min at 4°C. After permeabilization, ALI sections were incubated with blocking solution consisting of 10% goat serum and 0.1% (v/v) Triton X100 in PBS for 1 hours and then incubated with primary antibodies diluted in 1% BSA-PBS for 2 hours at room temperature. Appropriate fluorescence-conjugated secondary antibodies from goat were incubated for 1 hour. DNA was stained with Hoechst 33342 and mounted with Fluoromount-G (Thermo Scientific 00-4958-02) mounting media. ALI sections were examined under a Leica SP8 confocal microscope. The primary and secondary antibodies used in this study include Keratin 13 (Abcam; ab16112), Keratin 5 (Abcam; ab64081) Keratin 16 (Abcam; ab76416), S100A7 (Abcam; ab13680), Integrin β1 (Santa Cruz; sc-18887), and CD98 (Santa Cruz; sc-21745).

### qRT-PCR of KSHV-infected EpiOral tissues

Total RNA was extracted with Tri reagent (Sigma Cat. T9424). 1 ug of RNA was incubated with DNase I (Sigma Cat. AMPD1) and reverse transcribed using the iScript cDNA Synthesis Kit (Bio-Rad Cat. 1708891). The resulting cDNA was used for qPCR with SsoAdvanced Universal SYBR Green Supermix (Bio-Rad Cat. 1725270). Primer sequences used for qRT-PCR are found in S2 Table.

### Measuring KSHV genome copy numbers

Supernatant collected at indicated time point were treated with DNase to remove any non-encapsidated viral DNA. DNase was inactivated and viral capsid are disrupted by heating samples at 95°C for 30 minutes. BAC16 bacmid was used for KSHV genomic DNA standard. Twenty nanograms of DNA was used for qPCR with SsoAdvanced Universal SYBR Green Supermix (Bio-Rad 1725271). qPCR assay used KSHV ORF11. Primer sequences are found in S2 Table.

### Preparation of EpiOral tissues for single-cell RNA sequencing

Mock- and KSHV-infected tissues were harvested at 3 days and 6 days post infection. Tissue inserts were removed from the 6-well plate and placed into a new 6-well plate with enough PBS to coat the bottom. The inside of the insert was then washed with PBS to collect the supernatant. The tissues where then carefully separated from the insert membrane and chopped into small pieces in DMEM. The cells are then incubated for 40 minutes in 2 mL of 1 mg/mL collagenase and dispase (Roche Cat. 10269638001) in a shaking incubator set to 37°C and 250 rpm. The collagenase and dispase are then inactivated by adding 2 mL of DMEM and 10% FBS. The digested cells are then passed through a 0.45 um cell strainer and then washed with cold PBS with 0.04% BSA (ThermoFisher Cat. AM2618). The filtered cells are also passed through a flowmi cell strainer. Cells are counted with Invitrogen Countess II (ThermoFisher Cat. A27977).

### Single-cell RNA sequencing

For this study, three separate infections were used: the first two infection studies used the 10X Genomics Single Cell Gene Expression v2 kit and the third infection study used the v3 kit for the generation of single cell RNAseq libraries. Mock- or KSHV-infected tissues were digested into a single cell suspension and then immediately used for single cell RNA sequencing. Sample preparation and library generation were done following the v2 Rev E (CG00052), v3 Rev A (CG000183). After the libraries were generated, library quality was checked on an Agilent Bioanalyzer 2100 and a low read depth run on an Illumina MiSeq V2 flowcell was used to determine the number of reads required for library sequencing. A larger sequencing run with an Illumina NovaSeq 6000 was then used to fully sequence single cell libraries. Sequencing and demultiplexing was handled by the University of Southern California Molecular Genomics Core. Sequencing data is publicly available at NCBI GEO (GSE184257).

### Data analysis of single-cell RNA sequencing

Initial processing of the fastq files was done using 10X Genomics Cell Ranger v3.1. A custom reference was made that combined the human hg38 reference genome, KSHV JSC-1 reference genome (GQ994935.1), GFP, and RFP transcripts. The resulting filtered gene matrices from Cell Ranger was used for further analysis with Seurat v3 and R (version 4.1.1). Initial processing of the fastq files was done using 10X Genomics Cell Ranger v3.1. A custom reference was made that combined the human hg38 reference genome, KSHV JSC-1 reference genome (GQ994935.1), GFP, and RFP transcripts. The resulting filtered gene matrices from Cell Ranger was used for further analysis with Seurat v3 and R (version 4.1.1). Cells with a mitochondrial read percentage greater than 10% or expressing less than 200 genes were excluded from analysis (S4 Fig). To account for potential batch effects between the three sets of data, we used the sctransform [47] and glmGamPoi [48] packages to normalize and stabilize variance in the data.

To annotate the epithelial layer of each cell, we chose gene expression markers that have been previously reported [10–16]. Canonically, there are four layers in the oral epithelia: basal, spinous, granular, and cornified. From our data, we found it difficult to differentiate between the spinous and granular layers, so these two layers were combined and classified as the intermediate layer. Basal cells were identified by enrichment of *KRT5*, *KRT14*, and *S100A2*. Intermediate cells were identified by enrichment of *NOTCH3*, *S100A4*, and *KRT13*, and *TGM5*. Superficial layer cells were identified by enrichment of *S100P*, *SPRR2A*, and *SPRR2E*. (S4 Fig.).

KSHV-infected cells were identified by expression of GFP transcripts. If cell was GFP+ and PAN+ or RFP+, cell was annotated as a lytic cell. If cell was GFP+, RFP-, and PAN-, cell was categorized as a latent cell. Latent-2 cell was identified as GFP+ cell with K2, K3, or K4 expression.

Differentially expressed genes were identified using the FindMarkers function with a cutoff of p-value < 0.05 and |fold change| > 1.5. Gene ontology was performed on the genes using either the PANTHER Classification System [49,50] or Qiagen’s Ingenuity Pathway Analysis (QIAGEN Inc., https://digitalinsights.qiagen.com/IPA) [51]. Bar charts were generated with Prism 9.0.0 (Graphpad Software, LLC). Pathway bubble plots were generated with the ggplot2 package [52] and heatmaps were generated with pheatmap (https://cran.r-project.org/web/packages/pheatmap/index.html).

## Supporting information

S1 Fig rKSHV.219 infection of 3D epithelial tissues. (A) (Left) Schematic of light and deep wounding in the 3D epithelial tissues. (Right) H&E-stained tissue sections of lightly scored and deeply scored 3D epithelial tissues infected with rKSHV.219. Bars = 100 μm. (B) GFP and RFP expression upon rKSHV.219 infection in 3D epithelial tissues at indicated time point. GFP indicates KSHV-infected cells and RFP indicates KSHV lytic cells. The images are taken from the top of 3D cell cultures. Bars = 400 μm. (C) Immunofluorescence images of KSHV-infected tissues at 6 days post infection. GFP and RFP represent KSHV-infected cells and KSHV lytic cells, respectively. ORF59 or LANA staining is purple and Hoechst DNA-stained nucleus are shown in blue. Bars = 25 μm. (D) Immunofluorescence images of 3D epithelial tissues. Integrin β1 and CD98 are stained in red. Hoechst DNA-stained nucleus are shown in blue. Bars = 100 μm.
(TIFF)

S2 Fig FACS of KSHV-infected 2D and 3D tissues. Representative FACS plots showing the percentage of GFP+ and RFP+ in 2D HOKg cells or 3D epithelial tissues infected with rKSHV.219 at the indicated timepoints. Mock-infected sample was at 3 dpi.
(TIFF)

S3 Fig Fluorescence images of KSHV infected oral epithelial ALI culture. (A) GFP and RFP expression in cells infected with rKSHV.219 for 1, 3, 6, and 9 days in 3D epithelial tissues. The images are taken from the top of 3D cell cultures. Bars = 200 μm. (B) Immunofluorescence images of ORF59 or K8.1 (green), LANA (red), and Hoechst (blue) in KSHV-infected tissues at 6 dpi. White arrows indicate LANA+ cells and green arrows indicate lytic cells. Bars = 25 μm. (C) Immunohistochemistry of K2 or K3 (green), LANA (red), ORF 59 (purple), and Hoechst (blue) in KSHV-infected tissues at 6 dpi. White arrows indicate LANA+ cells and green arrows indicate latent-2 cells. Bars = 25 μm.
(TIFF)

S4 Fig Quality Control of Single Cell RNAseq. Violin plots showing the number of genes (left), the number of transcripts (middle) and the percent of mitochondrial gene (right) based on (A) cells at different time points, (B) KSHV lifecycle, or (C) epithelial layer.
(TIFF)

S5 Fig Dotplot of Epithelial Differentiation Marker Expression. Dotplot showing the expression of epithelial layer gene markers for each cluster of cells identified by Seurat. Based upon marker expression, each cluster was annotated as either basal, intermediate, or superficial.
(TIFF)

S1 Table Infected cell types at each time point. Table of non-infected, latent, latent-2, and lytic cell types in mock- or KSHV-infected tissues at 3 and 6 dpi.
(TIFF)

S2 Table Primers used for qPCR assays. Table of forward and reverse primers used for qPCR assays used in Fig 1.
(TIFF)
