# Supplementary figures and images for: Single-cell analysis of Kaposi’s sarcoma-associated herpesvirus infection in three-dimensional air-liquid interface culture model
Source: PLoS Pathog. 2022 Aug 17;18(8):e1010775. doi: 10.1371/journal.ppat.1010775 (PMC9385030; doi:10.1371/journal.ppat.1010775)

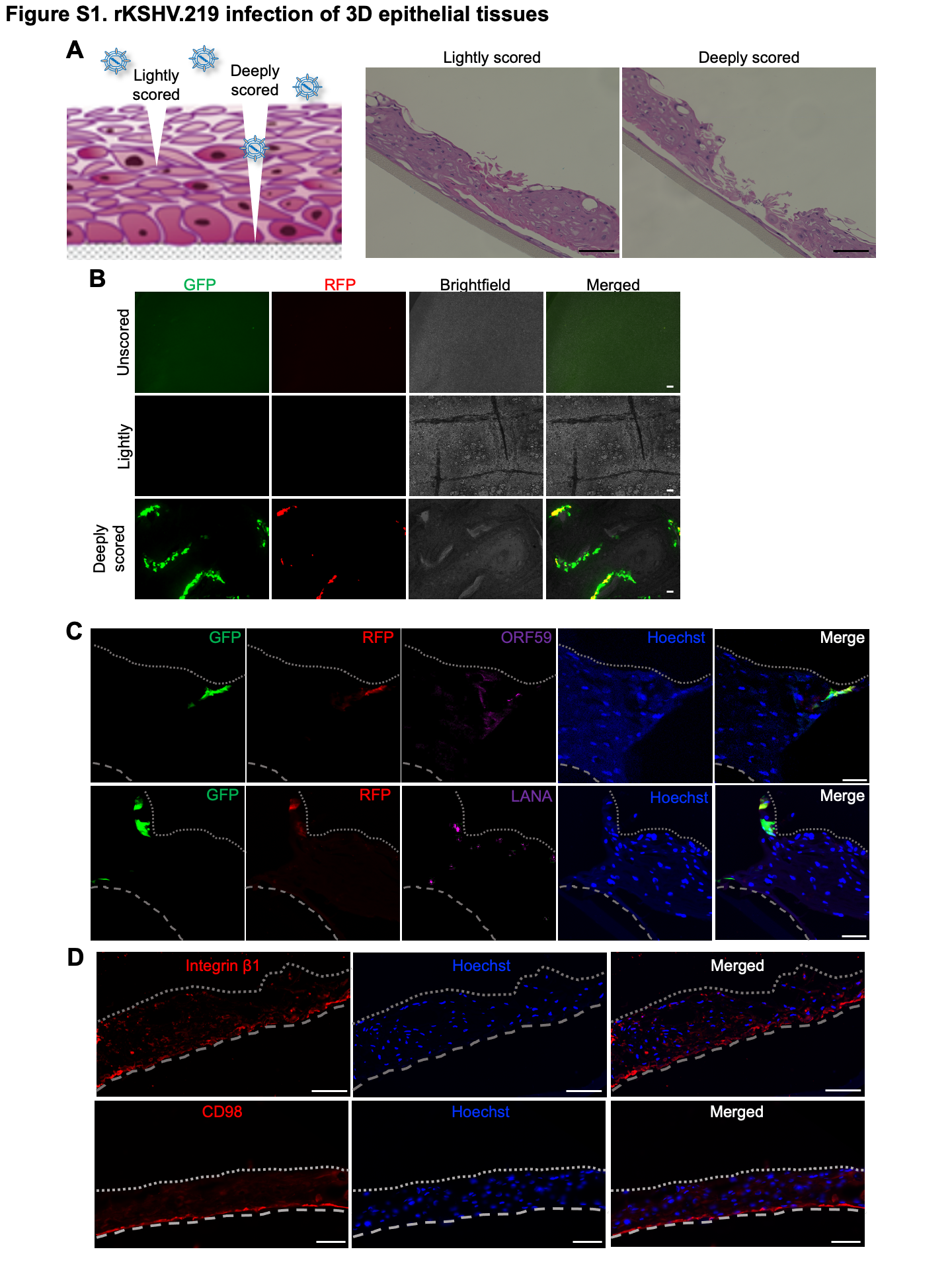

Supplement: S1 Fig — (A) (Left) Schematic of light and deep wounding in the 3D epithelial tissues. (Right) H&E-stained tissue sections of lightly scored and deeply scored 3D epithelial tissues infected with rKSHV.219. Bars = 100 μm. (B) GFP and RFP expression upon rKSHV.219 infection in 3D epithelial tissues at indicated time point. GFP indicates KSHV-infected cells and RFP indicates KSHV lytic cells. The images are taken from the top of 3D cell cultures. Bars = 400 μm. (C) Immunofluorescence images of KSHV-infected tissues at 6 days post infection. GFP and RFP represent KSHV-infected cells and KSHV lytic cells, respectively. ORF59 or LANA staining is purple and Hoechst DNA-stained nucleus are shown in blue. Bars = 25 μm. (D) Immunofluorescence images of 3D epithelial tissues. Integrin β1 and CD98 are stained in red. Hoechst DNA-stained nucleus are shown in blue. Bars = 100 μm. (TIFF) [file ppat.1010775.s001.tiff]

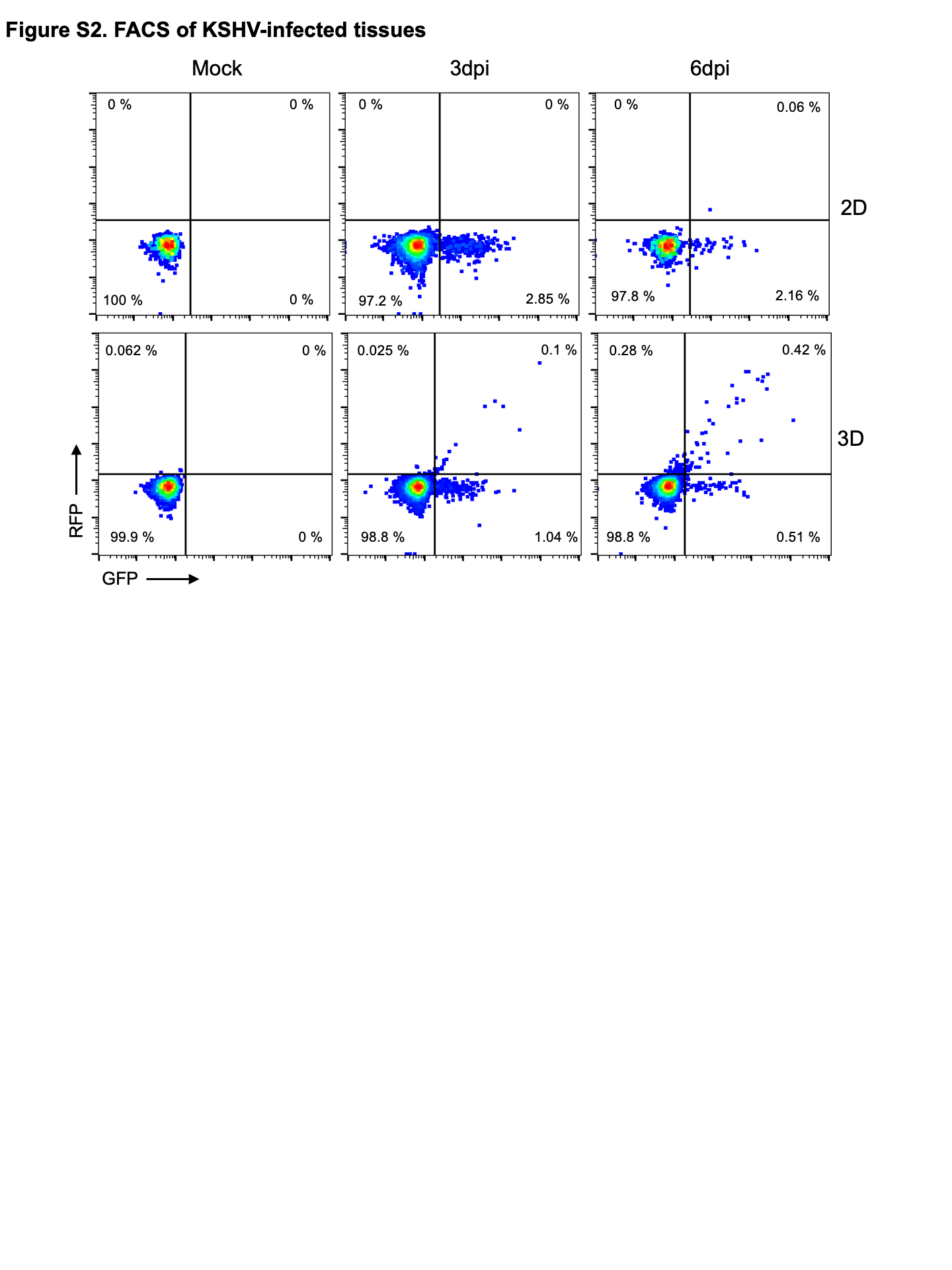

Supplement: S2 Fig — Representative FACS plots showing the percentage of GFP+ and RFP+ in 2D HOKg cells or 3D epithelial tissues infected with rKSHV.219 at the indicated timepoints. Mock-infected sample was at 3 dpi. (TIFF) [file ppat.1010775.s002.tiff]

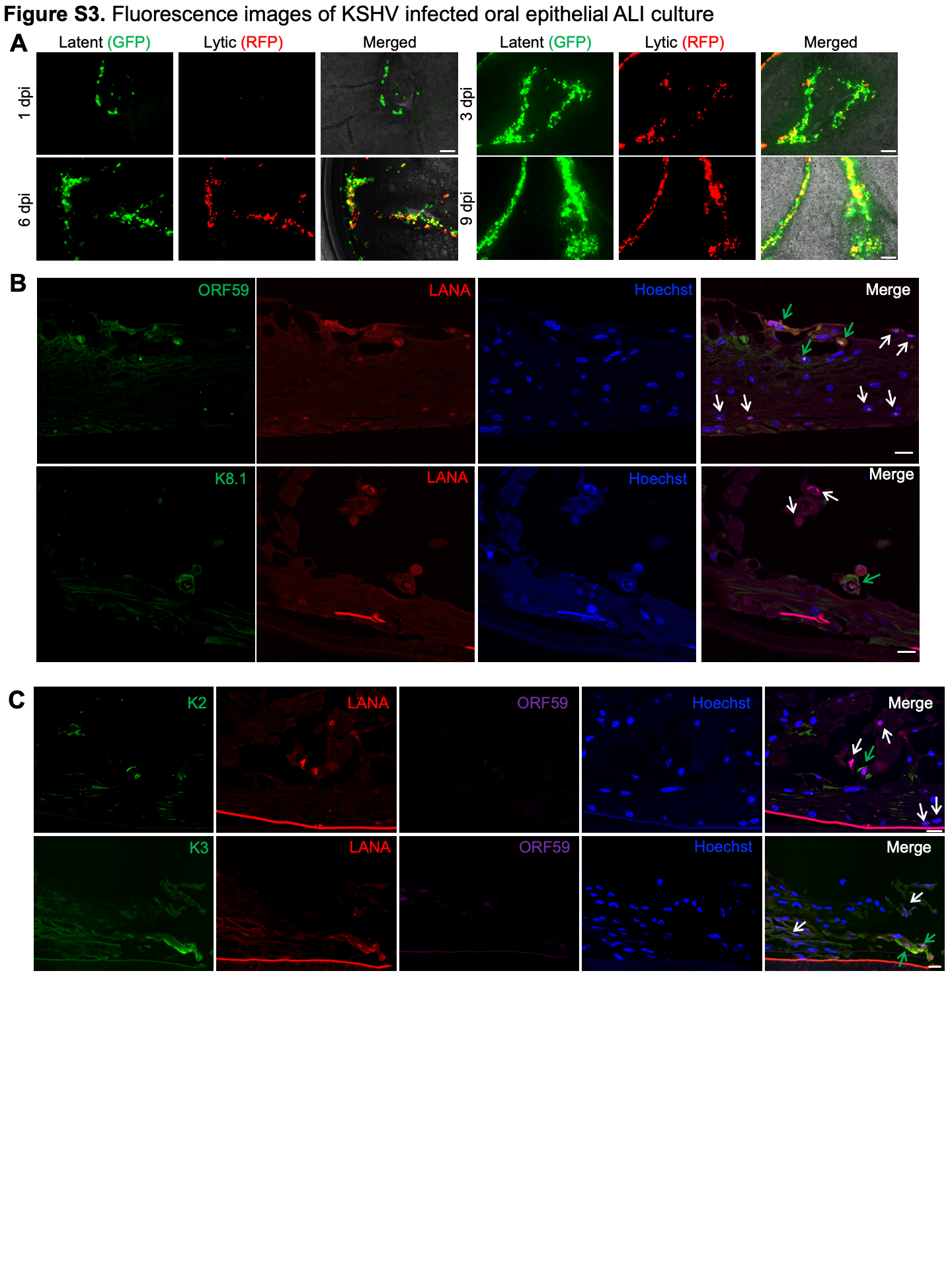

Supplement: S3 Fig — (A) GFP and RFP expression in cells infected with rKSHV.219 for 1, 3, 6, and 9 days in 3D epithelial tissues. The images are taken from the top of 3D cell cultures. Bars = 200 μm. (B) Immunofluorescence images of ORF59 or K8.1 (green), LANA (red), and Hoechst (blue) in KSHV-infected tissues at 6 dpi. White arrows indicate LANA+ cells and green arrows indicate lytic cells. Bars = 25 μm. (C) Immunohistochemistry of K2 or K3 (green), LANA (red), ORF 59 (purple), and Hoechst (blue) in KSHV-infected tissues at 6 dpi. White arrows indicate LANA+ cells and green arrows indicate latent-2 cells. Bars = 25 μm. (TIFF) [file ppat.1010775.s003.tiff]

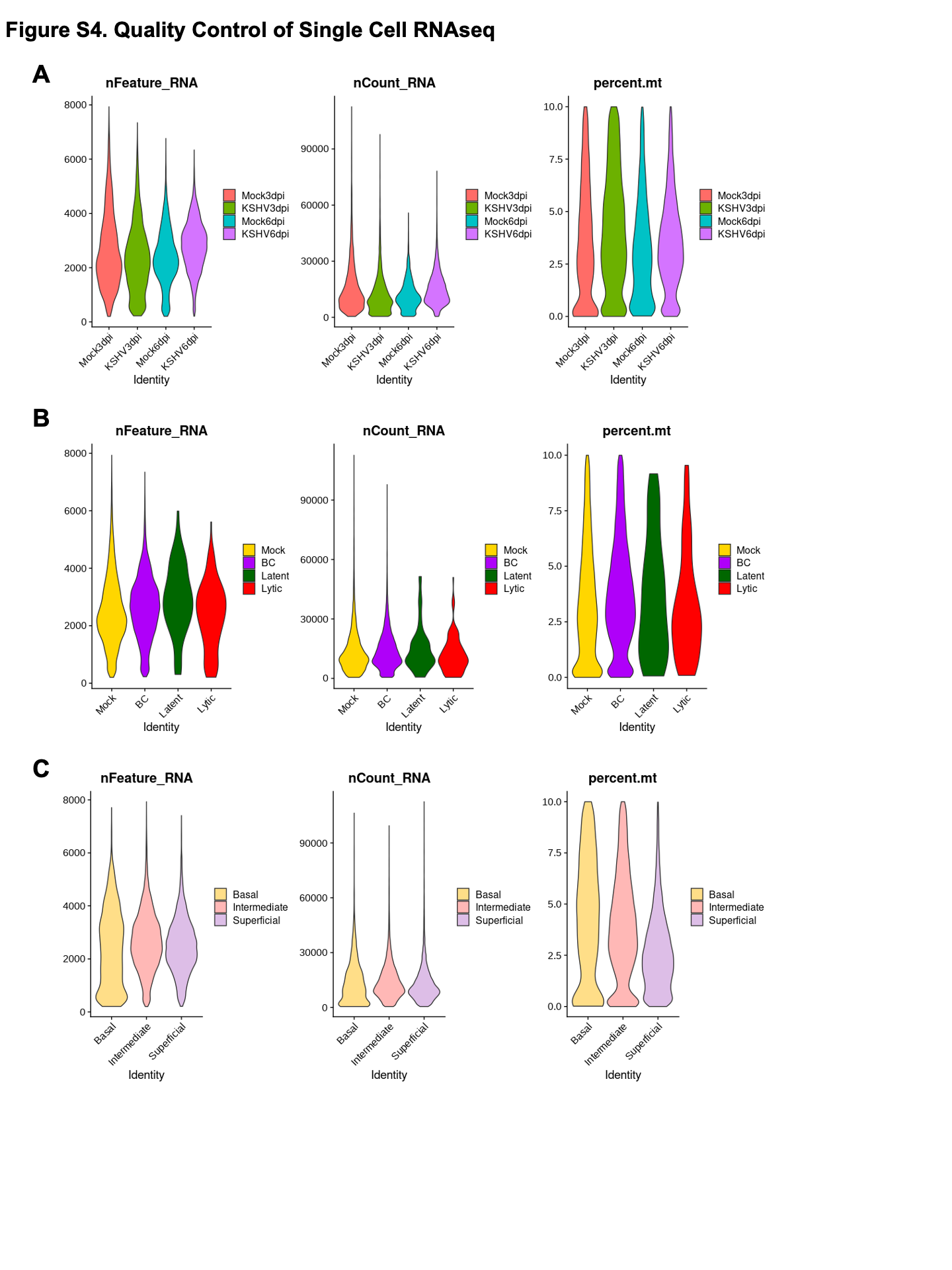

Supplement: S4 Fig — Violin plots showing the number of genes (left), the number of transcripts (middle) and the percent of mitochondrial gene (right) based on (A) cells at different time points, (B) KSHV lifecycle, or (C) epithelial layer. (TIFF) [file ppat.1010775.s004.tiff]

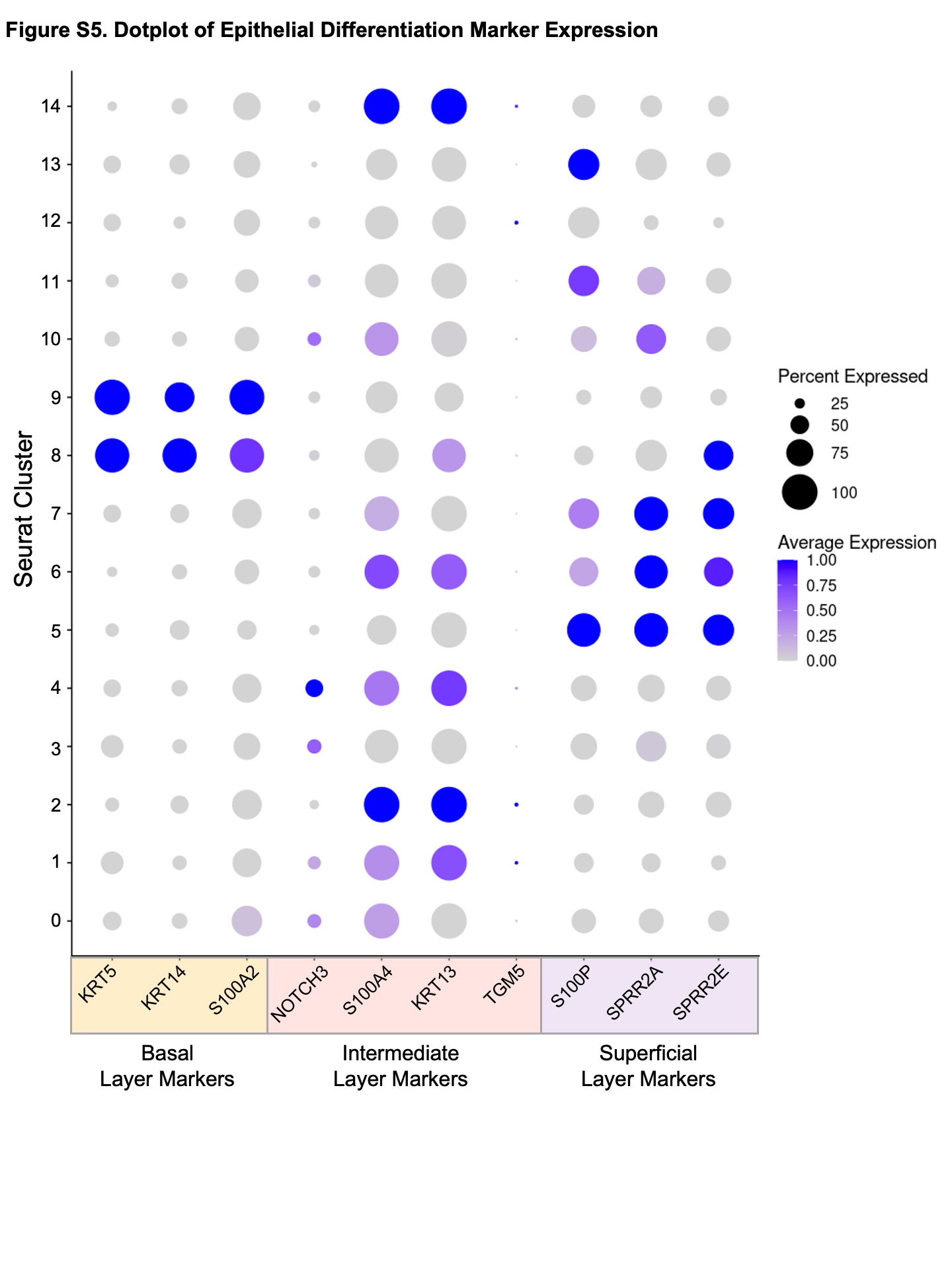

Supplement: S5 Fig — Dotplot showing the expression of epithelial layer gene markers for each cluster of cells identified by Seurat. Based upon marker expression, each cluster was annotated as either basal, intermediate, or superficial. (TIFF) [file ppat.1010775.s005.tiff]

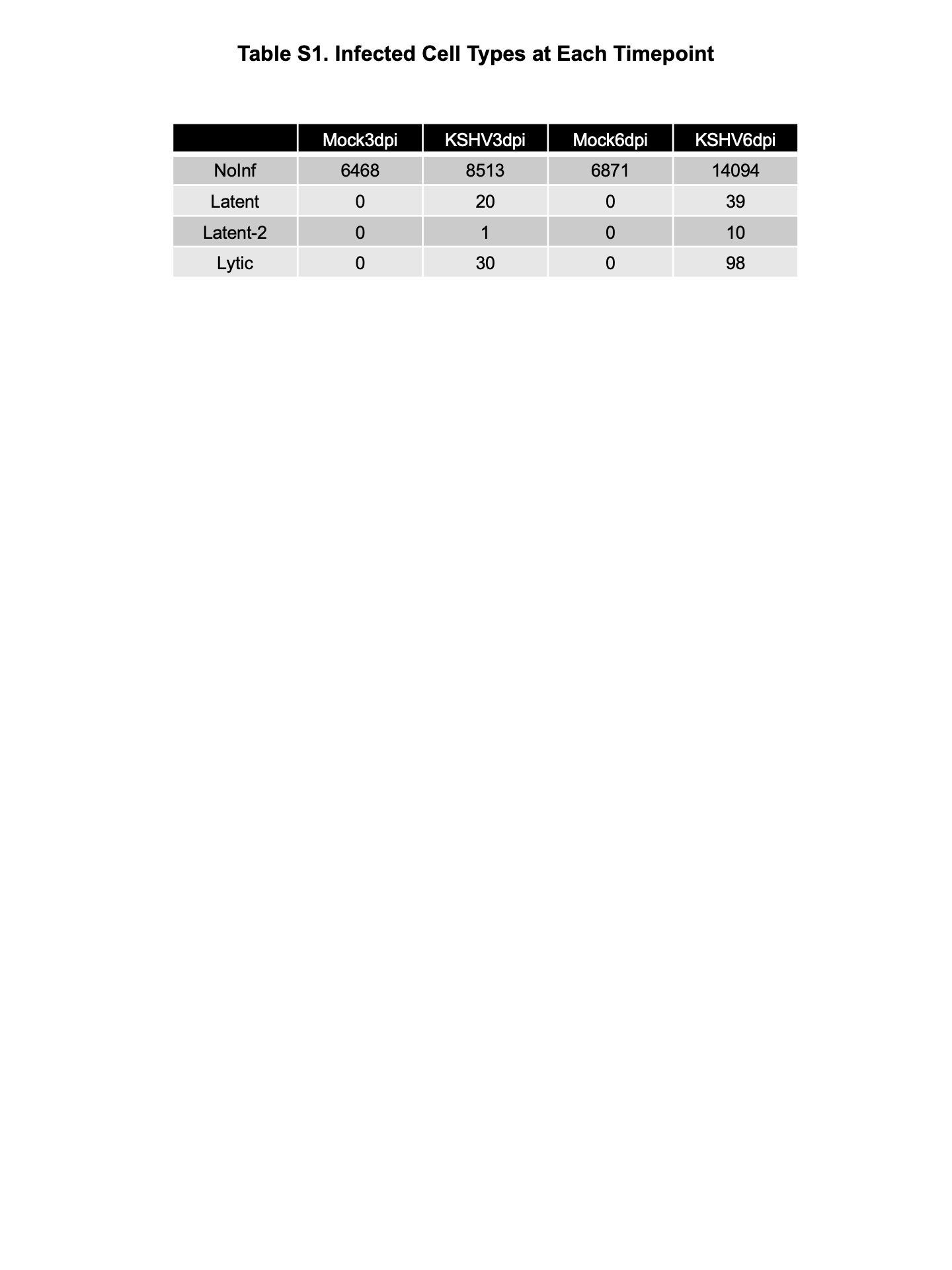

Supplement: S1 Table — Table of non-infected, latent, latent-2, and lytic cell types in mock- or KSHV-infected tissues at 3 and 6 dpi. (TIFF) [file ppat.1010775.s006.tiff]

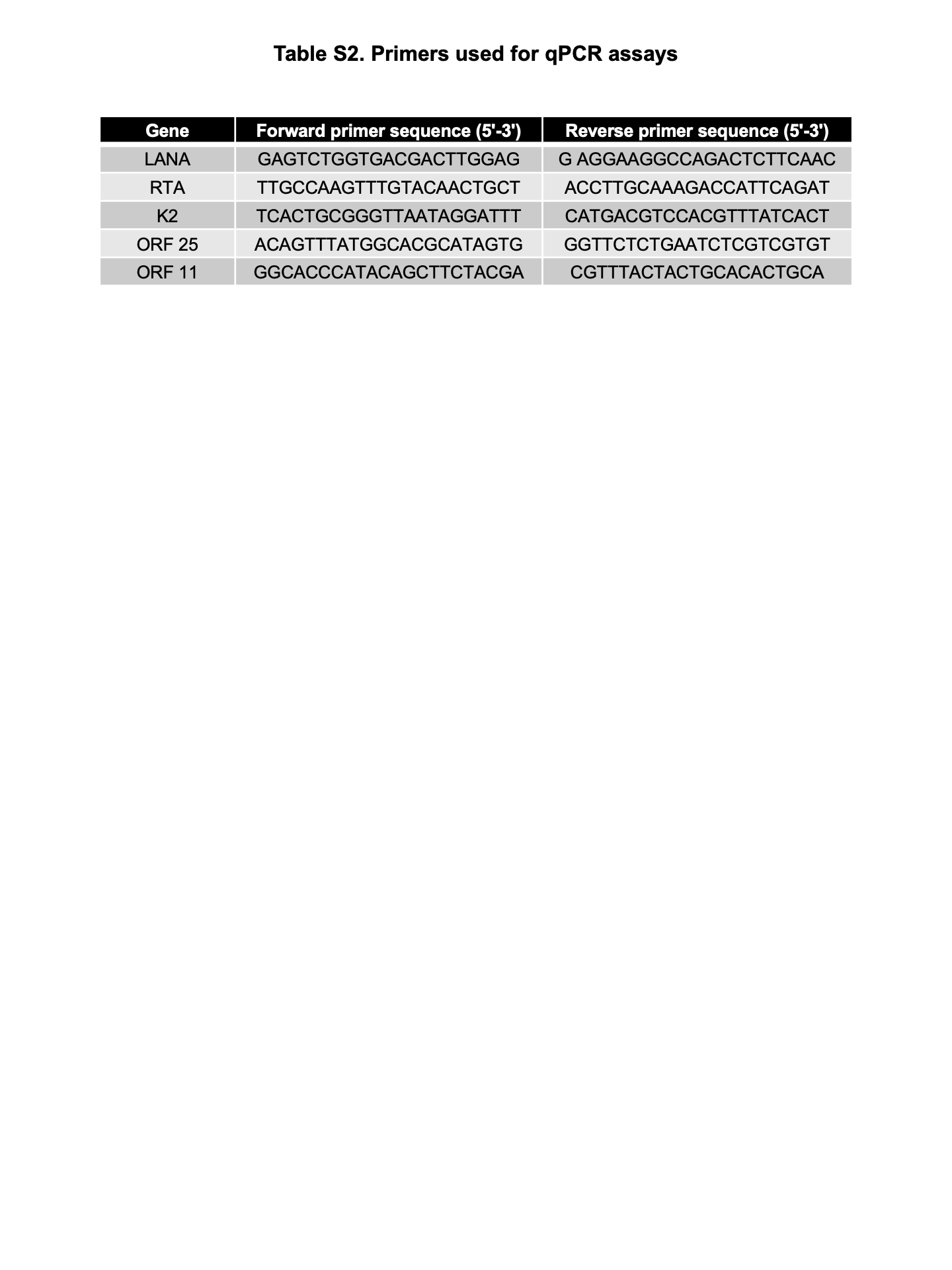

Supplement: S2 Table — Table of forward and reverse primers used for qPCR assays used in Fig 1. (TIFF) [file ppat.1010775.s007.tiff]
